# Oceanic feelings and their relationship to spirituality and personality organization

**DOI:** 10.3389/fpsyg.2025.1558537

**Published:** 2025-04-22

**Authors:** Sarah Straßnig, Tobias Herzl, Afrodita Latifi, Jürgen Fuchshuber, Paul Jimenez, Human-Friedrich Unterrainer

**Affiliations:** ^1^Institute of Psychology, University of Graz, Graz, Austria; ^2^Division of Medical Psychology, Psychosomatics and Psychotherapy, Medical University of Graz, Graz, Austria; ^3^Department of Psychoanalysis and Psychotherapy, Medical University of Vienna, Vienna, Austria; ^4^Comprehensive Center for Clinical Neurosciences and Mental Health, Medical University of Vienna, Vienna, Austria; ^5^Center for Integrative Addiction Research, Grüner Kreis Society, Vienna, Austria; ^6^Department of Religious Studies, University of Vienna, Vienna, Austria; ^7^Division of Psychiatry and Psychotherapeutic Medicine, Medical University of Graz, Graz, Austria; ^8^Institute of Psychotherapy Science, Sigmund Freud University Vienna, Vienna, Austria

**Keywords:** affective neuroscience, connectedness, oceanic feelings, schizotypy, spirituality, personality organization

## Abstract

**Background:**

Oceanic feelings, which reflect a sense of boundlessness, unity but also fragmentation can evoke spiritual experiences and possibly lead to psychosis proneness. This study explores oceanic feelings psychometrically, by examining their connections with spirituality, schizotypy, and personality organization.

**Methods:**

A sample of 480 adult non-clinical participants (66.2% female) completed an online survey, which included the Oceanic Feeling Scale, the MI-RSWB-18, SPQ-B, and the IPO-16. All relationships were evaluated in a path analysis to assess the direct and indirect effects of positive and negative oceanic feelings on schizotypy, connectedness, and general religiosity, with personality organization as a mediator.

**Results:**

Positive oceanic feelings were strongly correlated with increased connectedness (*r* = 0.68) and general religiosity (*r* = 0.61). We measured weak to moderate associations of negative oceanic feelings with schizotypy (*r* = 0.41) and personality organization (*r* = 0.39). Path analysis revealed that personality organization mediated the relationship between negative oceanic feelings and schizotypy (*β* = 0.11, *p* < 0.001). Additionally, the relationship between negative oceanic feelings and general religiosity was also fully mediated by personality organization (*β* = 0.05, *p* < 0.011). The association between negative oceanic feelings and connectedness was partially mediated by personality organization (*β* = 0.07, *p* < 0.001).

**Discussion:**

The findings reveal a dual role of oceanic feelings: positive oceanic feelings enhance general religiosity and connectedness independently of personality organization, while negative oceanic feelings are mediated by personality dysfunction, influencing schizotypy and spirituality. Strengthening personality organization could serve as a protective factor against the destabilizing effects of negative oceanic experiences, offering insights for psychotherapy and spiritual counselling.

## Introduction

*“The dividing line between madness and mysticism is the same as that between depth and height. The same experience that can lead one person to the depths of despair can carry another to the heights of realization.” (analogous to Laing,* *1967**)*

Many people report experiences where the line between ordinary perception and a heightened awareness blurs—moments of deep connection, expanded consciousness, or loss of self (Hofmann, 2017). Contrary to popular belief, such mystical experiences are common; research shows that 52–100 percent of people may have these at some point (Hoffmann and Heise, 2017). These experiences often involve “oceanic feelings,” characterized by a sense of boundary dissolution, spaciousness, or oneness with a higher power, like God or the universe (Schmautz et al., 2024). While this feeling often brings peace and connection, the breakdown of personal boundaries can sometimes cause discomfort, fear, or loneliness (Saarinen, 2014).

### Psychosis and spirituality

Until recently, psychosis and spirituality were largely separated, with spirituality often marginalized in scientific and clinical fields (Machado and Moreira-Almeida, 2021). However, a growing number of researchers and organizations, like the U.K.’s Spiritual Crisis Network, now highlight the link between spirituality and psychosis. Psychotic and spiritual experiences differ in interpretation but share a crossing of typical cognitive and emotional boundaries (Clarke, 2010). Many cultures describe spiritual phenomena resembling psychotic symptoms, such as spiritual crises experienced by saints and mystics, including experiences like hallucinations and delusions. Margery Kempe, a medieval mystic, exemplifies this overlap; her intense visions of Christ led her to live a radically devout life. She embarked on chaotic pilgrimages, often barefoot and without material comforts, expressing herself loudly and emotionally with public screams. Today, her behavior might be interpreted as falling within schizophrenia or manic spectrum disorders (Windeatt, 1985; Clarke, 2010).

### Mystical experiences

In *The* Var*ieties of Religious Experience*, William James outlines four characteristics of mystical experiences. First, they are inexpressible—beyond words and directly felt, more emotional than intellectual, making them hard to communicate to those without similar experiences. Second, mystical experiences have a “noetic quality,” providing insights into spiritual realities that feel authoritative and profoundly meaningful. The third characteristic is their brief duration, typically lasting from half an hour to 2 h, though these experiences can be recognized if they recur. Finally, passivity defines mystical experiences; they occur outside the person’s control, as if a greater power were taking over. These experiences are not mere interruptions of daily life but create a lasting sense of meaning that can endure for weeks or even a lifetime (James, 1902).

Historically, people often interpreted unusual experiences as spiritual or mystical visions. In ancient Greece, for example, epileptic seizures were seen as a “sacred illness” with mystical significance. With modern neuroscience, however, mystical states and hallucinations are frequently viewed as neurological disorders or signs of mental illness. This shift raises questions about the spiritual meaning of such experiences, showing how cultural and scientific paradigms shape our understanding of mysticism. Traditional scientific models, starting with Newtonian mechanics in the 18th century, largely ignored consciousness, focusing only on objective, measurable facts.

Neurophysiological research suggests that mystical experiences can arise from meditation, hallucinogens, or neurological conditions like temporal lobe epilepsy. These states or experiences are observed in both healthy individuals and those with psychotic disorders, indicating a basic capacity of the human brain for mystical experience. Mystical experiences also occur in pathological states like psychosis and epilepsy, suggesting that the brain employs specific mechanisms for these states in both health and illness (Fenwick, 2010). Primary emotional systems may offer some insight into how mystical experiences arise, and in turn, shape behavior and personality (see Panksepp and Biven, 2012; Solms and Friston, 2018).

### Primary emotions

In Jungian psychology, the “self” is viewed as the core of personality, with physiological foundations possibly rooted in the brainstem, the brain’s central organizing system (Alcaro et al., 2017). While historically significant for its role in shaping early understandings of brain-behavior relationships, the subcortical midline structures (SCMS), along with the limbic system, are crucial for generating and regulating basic emotional states. These structures, which connect the brain to the spinal cord and body, are fully developed at birth and are evolutionarily conserved across species ranging from reptiles to mammals (MacLean, 1990; Wiest, 2012). Studies in humans and animals show that damage to the SCMS can lead to a coma-like state where mental activity halts, with organisms showing only basic life signs but no purposeful action, like eating, sleeping, procreating and play. Destruction of the periaqueductal grey matter (PAG) at the SCMS’s center eliminates self-related processing, leaving only minimal alertness without environmental awareness (Panksepp and Biven, 2012). Evolutionarily, the SCMS houses neural systems that drive instinctive behaviors vital for survival and reproduction. This foundational emotional structure, closely tied to the SCMS, can be found as termed the “affective core self” in the literature (Alcaro et al., 2017).

Contemporary neuroscience highlights that personality and psychological functions arise from the intricate interactions between cortical and subcortical regions. For example, adaptive behavior depends on the coordinated processing of information across neural networks. The prefrontal cortex plays a crucial role in modulating the amygdala’s activity, thereby shaping emotional responses according to situational demands (Ochsner and Gross, 2005). This underscores the idea that psychological processes emerge from network dynamics rather than isolated brain structures (Friston, 2011). Moreover, personality traits and attitudes are increasingly conceptualized as latent features of these network interactions, aligning with perspectives on functional and effective connectivity (Sporns and Betzel, 2016).

As revealed by brain stimulation research, SCMS are linked to seven primary emotional systems (Panksepp, 2011). In Affective Neuroscience, Panksepp (1998) classified these into four positive and three negative systems: SEEKING, CARE, PLAY, and LUST as positive systems, and SADNESS, ANGER, and FEAR as negative ones. The SEEKING system drives energy and exploration, aiding in the search for food and mates; CARE promotes nurturing behavior; PLAY supports social bonding and motor skills; and LUST underpins reproductive behavior. The negative systems include SADNESS, which responds to social loss, ANGER, which encourages defense of territory and offspring, and FEAR, which triggers escape responses to danger (Panksepp, 2011). Notably, besides the primary affective systems, Panksepp identified spirituality as one of humanity’s higher emotions, distinguishing it from basic emotional systems by its role in meaning-making and transcendence. His model suggests that spirituality emerges from the interaction of core affective systems, potentially offering an evolutionary advantage by providing life with meaning and inner strength (Davis et al., 2003; Davis and Panksepp, 2011). Throughout history, spiritual and religious practices have played a fundamental role in shaping human societies (Atran, 2002).

Research using lesion studies identified a neural network linked to self-transcendent mystical experiences, with the periaqueductal grey (PAG) acting as the central hub (Ferguson et al., 2021). Since the PAG also plays a key role in generating the seven primary emotions, oceanic feelings might serve as the emotional foundation of spirituality (Asp, 2022). However, other studies have suggested that personality traits may not only be influenced by the connectivity between different brain regions but also by specific patterns of neural activity and their interactions. For instance, some research indicates that certain personality characteristics could be associated with regional differences in neural excitability or synaptic density, further highlighting the complexity of the neurobiological foundations of personality (Derome et al., 2020). Emotional states are dynamic brain and body patterns that influence behavior and trigger specific neuronal activations, guiding thought and action. When these states spread to the spinal cord and other physical systems, they provoke instinctive reactions on a behavioral level. As they reach higher brain regions, they may appear as mental images, thoughts, or representations, which, in Jungian terms, align with archetypes (see Alcaro et al., 2017 for further discussion). In correspondence to this notion, we hypothesize that oceanic feelings, arising in the PAG, develop into mental representations, such as oceanic states, which can manifest as spiritual connectedness or, conversely, as psychotic disturbance.

### Oceanic feelings

The concept of an oceanic feeling emerged from a correspondence between the French dramatist Romain Rolland and the father of psychoanalysis Sigmund Freud. Rolland asked Freud to analyze a sensation he believed was the source of all religions: the “feeling of the ‘eternal’”—a limitless, oceanic sense of unity (Parsons, 1999, p. 173). Rolland described it as a spontaneous, renewing phenomenon, independent of organized religion and experienced by countless individuals in varying forms. Freud interpreted it as a regression to an infantile state of unity between infant and mother, linked to a desire to restore boundless narcissism. He argued that this feeling lacked intrinsic religious significance (Saarinen, 2014). Furthermore, the sea symbolizes the oceanic feeling, embodying both discovery and risk. Historically feared as an uncontrollable force, attitudes shifted in the 19th century, when the sea became a place of reflection and awe, offering encounters with the divine or self. Romantic writers like Jules Michelet celebrated its power and inspiration, while others, such as Herman Melville, explored its darker, perilous depths. This duality of the oceanic feeling—balancing danger with discovery, intrigue with anxiety—illustrates the sea’s enduring symbolic resonance (Holtorf, 2014). Accordingly, Saarinen (2012) outlined three main descriptions of the oceanic state. (1) Romain Rolland regarded it as a metaphysical experience, merging with the universe in a spiritual feeling. (2) Sigmund Freud viewed it as rooted in childhood, reflecting the infant’s sense of unity with the mother and a return to an original state. (3) Anton Ehrenzweig offered a cognitive-perceptual perspective, describing it as a dissolution of boundaries between conscious and unconscious perceptions, particularly relevant in creative processes. Correspondingly, Saarinen (2014) describes oceanic feelings as episodes of temporary self-boundary dissolution or lasting states of unity, openness, and immanence. Often positive, these feelings range from liberating to frightening, evoking either a meaningful connection to the universe or a terrifying, isolating, and nihilistic outlook on life.

The way we perceive the world may be influenced by oceanic feelings, though the extent of this influence likely varies among individuals. Positive experiences foster connection and unity with the universe, while negative ones evoke a sense of threat, blurred boundaries, and dissolution into vastness. The negative perspectives are influenced by feelings of lack and loss, often associated with mental disorders. Such conditions can lead to isolation, a diminished sense of meaning, emotional detachment, and impaired relationships. The inability to build connections is particularly distressing, compounded by reduced personality functioning, including difficulty regulating emotions, and by withdrawal from social interactions (Saarinen, 2014; Sarkar and Adshead, 2006). The oceanic feeling has both positive and negative dimensions. Positive feelings reflect oneness with something greater, like a higher power influencing life or the universe. Negative feelings involve despair, a sense of crumbling within, or experiences akin to hell (Schmautz et al., 2024). Holtorf (2014) highlights the ambivalence of this feeling, with the sea symbolizing both the promise of self-expansion and the threat of losing oneself to its depths.

### Schizotypy and schizophrenia

Studies on schizotypal traits suggest a spectrum ranging from personality differences to severe psychotic symptoms (Nelson et al., 2013). These traits often involve interpersonal difficulties, cognitive-perceptual issues, and disorganized features, partly influenced by genetics and environmental factors affecting brain development (Raine, 2005). Cognitive-perceptual traits include unusual beliefs and perceptions, while interpersonal difficulties involve social anxiety, emotional constraints, and limited relationships. The disorganized dimension includes idiosyncratic behavior and unstructured thoughts, often resulting in unusual speech (Raine, 1991). Two schizotypy categories have been mainly differentiated: neurodevelopmental, influenced by genetics and early environment, and pseudo-schizotypal traits linked to psychosocial challenges (Raine, 2005). Evidence supports a dimensional connection between schizotypy and schizophrenia, meaning that schizotypal traits exist on a continuum rather than as distinct categories (Nelson et al., 2013). This is further reinforced by family and adoption studies, which indicate that relatives of individuals with schizophrenia exhibit a higher prevalence of schizotypal traits, suggesting a genetic predisposition (Torgersen, 1985; Kendler et al., 1981). Genetic research confirms a shared susceptibility for both conditions (Fanous et al., 2007).

### Relationship between oceanic feelings, spirituality and schizotypy

**Figure 1:**
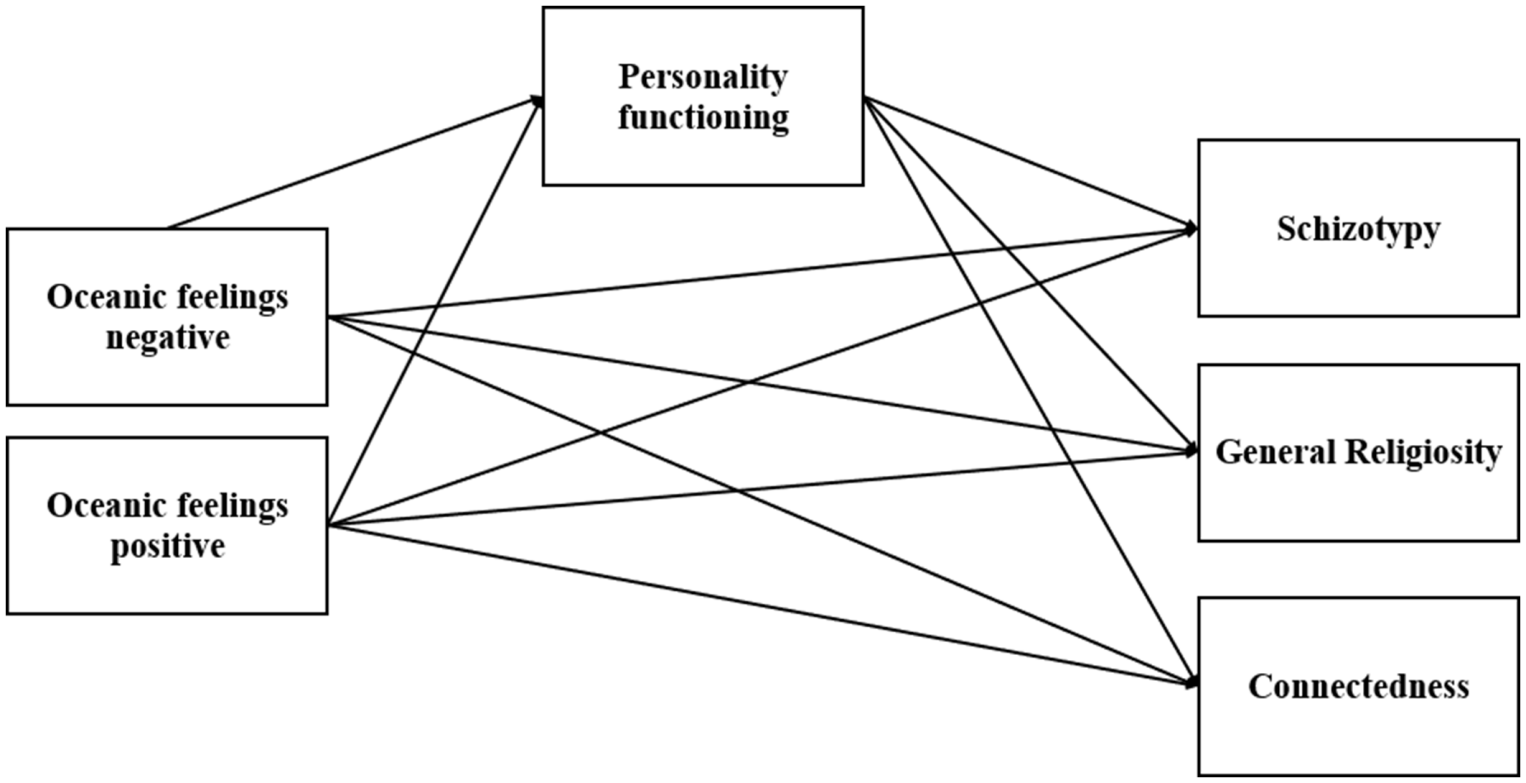
Explorative assumptions of the path model. Oceanic feelings and Schizotypy, General Religiosity, and Connectedness were model as correlated, however, the correlations are omitted in this figure for better clarity.

Oceanic feelings, characterized by experiences of unity or self-dissolution, have been proposed as a crucial link between spirituality and schizotypy, with both beneficial and detrimental effects on personality functioning and well-being (Unterrainer et al., 2011). Previous research suggests that spirituality within schizotypy is negatively associated with hope and forgiveness, often correlating with despair and social isolation. Conversely, it is positively related to connectedness, religiosity, and a sense of meaning (Unterrainer and Lewis, 2014). Cognitive-perceptual schizotypy strongly correlates with connectedness (Unterrainer and Lewis, 2014). Unusual experiences, a core aspect of schizotypy, predict engagement in modern spiritual practices (Farias et al., 2012). A religious or spiritual belief system may provide an interpretative framework for such unusual experiences, with individuals who exhibit stable personality structures being better equipped to integrate these experiences meaningfully (Schofield and Claridge, 2007). These findings suggest oceanic feelings shape schizotypy and spirituality in mystical experiences. Research on the relationship between personality organization, spirituality, and schizotypy remains limited (Willard and Norenzayan, 2017). Here, mystical experiences may strengthen personality in those with well-developed structures but weaken it in individuals with vulnerabilities or insufficient organisation (Feise-Mahnkopp, 2020). Claridge (2010) suggests that genetic factors, positive early life events, high intelligence, and a strong ego can protect against psychosis. This study aims to investigate the role of personality organisation as a mediating factor in the relationship between oceanic feelings, spirituality, and schizotypy. Based on the theoretical framework outlined above, we propose the following hypotheses:

1. Oceanic feelings are connected to general religiosity, connectedness and schizotypy.
2. The relationship between oceanic feelings and general religiosity, connectedness, and schizotypy is mediated by the level of personality organization. A proposed model posits that positive oceanic feelings contribute to enhanced personality organization, which in turn fosters greater connectedness, general religiosity, and adaptive aspects of schizotypy. In contrast, negative oceanic feelings may destabilize personality organisation, thereby increasing schizotypy (or psychosis proneness). Personality organization mediates the relationship between oceanic feelings and spirituality, encompassing general religiosity (institutional belief systems) and connectedness (experience-based faith beyond traditions). Positive oceanic feelings are linked to stronger personality organization, while negative feelings may destabilize and heighten schizotypy (see, e.g., Unterrainer, 2023 for an enhanced discussion)(Figure 1).

## Materials and methods

### Sample and procedure

Participants were recruited via personal networks, social media (Facebook, Instagram), public forums, and announcements at the University of Graz, Austria. They completed an online self-assessment survey after providing informed consent. Demographic data (age, gender, nationality, education, religious affiliation, occupation, psychiatric history, spiritual experiences) was collected, followed by standardized German questionnaires taking about 40 min. Some questionnaires were excluded as they pertained to a different research focus. Data was collected using LimeSurvey©, with participants remaining anonymous. Inclusion criteria required fluency in German and being over 18. Ethical approval was granted by the University of Graz Ethics Committee (No. 183–2023/24). Recruitment ran from July to October 2024. Written informed consent was given by all participants.

### Psychometric assessment

#### Oceanic feelings

Dispositions to oceanic feelings were measured with the OCEANIC Feelings Scale (Schmautz et al., 2024). The scale consists of 12 items rated on a 5-point Likert scale and provides scores for positive and negative oceanic feelings as well as a total score. Schmautz et al. (2024) report convincing internal consistencies for the instrument (OCEANic positive: *α* = 0.82; OCEANic negative: *α* = 0.88) Sample items include: “I had an experience in which my person seemed to merge with something larger” (positive) and ‘I once had the feeling of freezing up inside from loneliness’ (negative).

#### Dimensions religious/spiritual well-being

The Multidimensional Inventory of Religious/Spiritual Well-Being (MI-RSWB) was originally developed by Unterrainer et al. (2010) to different dimensions of subjective well-being as being related to religiosity/spirituality. The total score of the inventory is composed of six dimensions: Hope Immanent, Forgiveness, Experience of Sense and Meaning, Hope Transcendent, General religiosity and Connectedness. However, only the dimensions General religiosity and Connectedness were considered in the analysis (based on the 18-item short version of the scale; see Knorr et al., 2023). Marker items are used to illustrate the content of both scales, e.g., ‘I will be able to overcome all problems with God’s help.’ for General religiosity and ‘I have had experiences through which I have realized that nothing ever dies.’ for Connectedness.

#### Personality organization

The IPO-16 is a short version of the Inventory of Personality Organization (IPO), which is based on Kernberg’s psychodynamic model and was developed for self-assessment of the severity of personality disorganization, also serving as a predictor of personality disorders. It measures three central dimensions of personality organization: identity diffusion, primitive defense mechanisms and lack of reality testing. These dimensions reflect structural impairments relevant for the severity of personality disorders (see also Kernberg, 1984). As reported by Zimmermann et al. (2013), the IPO-16 has a high internal consistency (Cronbach *α* = 0.85) and has been validated in clinical studies. In this study, the total value of the IPO-16 is used for the measurement of the scale of personality organization. A higher value of personality organization reflects an increased probability of structural impairment or personality disorder.

#### Schizotypy

The Short Version of the Schizotypal Personality Questionnaire (SPQ-B) was developed by Raine and Benishay (1995) as a self-report instrument to assess the positive and negative aspects of schizotypy. It comprises three subscales covering cognitive perception, interpersonal relationships and disorganization (cf. Axelrod et al., 2001). Raine and Benishay (1995) report an adequate internal consistency of the three subscales with Cronbach’s α values ranging from 0.72 to 0.78.

### Data analysis

Using IBM SPSS Statistics 29, descriptive statistics and Pearson correlations were calculated, with two-sided testing for all *p*-values. Path analysis was conducted using IBM SPSS Amos 28 Graphics, utilizing personality organization (total score of IPO) as the mediator. The original model was refined by removing non-significant paths (*p* > 0.05), and model fit was evaluated using maximum likelihood estimation. The model is over-identified. Mediation and indirect effects were tested through bootstrap analysis, with a bias-corrected 95% confidence interval and 2000 bootstrap samples (Cheung and Lau, 2007). The following fit indices were used to assess excellent model fit: Root Mean Square Error of Approximation (RMSEA) ≤ 0.08 (CI: ≤0.1), Tucker-Lewis Index (TLI) and Comparative Fit Index (CFI) both ≥ 0.90, and χ^2^/df < 3 (Kline, 2015).

## Results

### Sample characteristics

In this study, 808 individuals participated online via LimeSurvey©. However, only 480 participants fully completed the test battery and provided clear, understandable responses which were included in the final data analysis. Additionally, only those who agreed to the informed consent form at the beginning of the study were considered. The sample was predominantly composed of 93.8% German-speaking individuals from Austria, Germany and Switzerland. Participants’ age ranged from 18 to 86 years, with an average age of 40.49 years (SD = 18.35). 66.2% of the participants identified as female, 31.3% as male and 2.5% felt they belonged to a different gender. A total of 51.0% of the participants owned a university degree at the time of their study participation, 34.8% completed their school-leaving certificate or A-levels, 12.1% completed vocational training or a technical college, and 2.1% either only completed compulsory schooling or had no qualifications at all. 7.7% reported having a diagnosed psychiatric disorder and 35.4% stated experience with drugs. Participants in the study reported an average life satisfaction of 7.23 (SD = 1.80), 10 indicating complete satisfaction and 0 complete dissatisfaction. 43.1% described themselves as spiritual or religious, while 24.6% declared they were somewhat spiritual or religious. 39.4% reported having had an experience where they felt that the boundaries between themselves and their environment were blurred. 48.1% asserted they had experienced an expanded state of consciousness and 45% reported a mystical experience in which they felt an intense sense of oneness with the universe, God, the divine, peace or love. In addition, 44.8% stated that they regularly practiced rituals, meditation or similar spiritual, religious or reflective practices. Further descriptive data are available in Table 1.

**Table 1:** Descriptive statistics: sociodemographic data and religious affiliation.

	*n*	%			*n*	%
Female	318	66.2	Education	University degree	245	51.0
Male	150	31.3		A-level	167	34.8
Different gender	12	2.5		Vocational training	58	12.1
Heterosexual	382	79.6		Compulsory schooling or have no qualifications	10	2.1
Homosexual	25	5.2	Confession	Catholic	220	46.0
Bisexual	46	9.6		Protestantic	64	13.1
Pansexual	10	2.1		Other Christian community	7	1.5
Other	17	3.6		Islamic	3	0.6
Single	196	40.8		Jewish	2	0.4
Married	139	29.0		Buddhist	3	0.6
Current relationship	106	22.1		Without profession of faith	19	4.0
Other	39	8.2		Left church	64	13.3
				Not belonging to any religion	96	20.0
				Other	2	0.4

n = Sample size; % = Percent.

### Correlations

Pearson correlations showed predominantly low correlations (according to Cohen, 1988) between positive and negative oceanic feelings with schizotypy (*r* = 0.24 to *r* = 0.29, all *p* < 0.01), and stronger correlations between general religiosity and connectedness (*r* = 0.61 to *r* = 0.68, all *p* < 0.01). Negative oceanic feelings showed a low correlation with connectedness (*r* = 0.13, *p* < 0.01) and moderate correlations with personality organization and schizotypy (*r* = 0.39 to *r* = 0.41, *p* < 0.01). Personality organization showed a low correlation with connectedness (*r* = 0.18, *p* < 0.01) and a strong correlation with schizotypy (*r* = 0.59, *p* < 0.01). General religiosity showed a strong correlation with connectedness (*r* = 0.65, *p* < 0.01) as well as a marginal correlation with schizotypy (*r* = 0.17, *p* < 0.01). Connectedness showed a small correlation with schizotypy (*r* = 0.27, *p* < 0.01). All correlations can be found in Table 2. All scales show good to excellent internal consistency (Cronbach’s *α* = 0.79 to 0.94), confirming the reliability of the measurements.

**Table 2:** Zero-order correlations for indicator variables.

	1. Oceanic feelings positive	2. Oceanic feelings negative	3. Personality organization	4. General religiosity	5. Connectedness	6. Schizotypy
Oceanic feelings positive	–					
Oceanic feelings negative	0.24**	–				
Personality organisation	0.09	0.39**	–			
General religiosity	0.61**	0.04	0.09	–		
Connectedness	0.68**	0.13**	0.18**	0.65**	–	
Schizotypy	0.29**	0.41**	0.59**	0.17**	0.27**	–
Mean values	2.85	2.57	2.07	2.73	2.68	2.53
Standard deviation	1.06	1.09	0.65	1.39	1.19	0.64
Cronbach‘s α	0.88	0.87	0.82	0.94	0.79	0.80

### Path analysis

Within a path model, the positive and negative oceanic feelings were correlated with each other and assessed for effects on connectedness, general religiosity and schizotypy. Based on the theoretical assumption personality organization was included as a potential mediator. The model was controlled for age. Before trimming, the model showed df = 0, which means it is just identified. Therefore, the Chi-square and fit indices cannot be meaningfully interpreted, as it fits perfectly by definition (Caughlin, n.d.). The only non-significant path is from positive oceanic feelings to personality organisation (*p* = 0.265). With the exception of the upper limit of the RMSEA, the final model showed good fit indices, resulting after trimming out the non-significant pathway: RMSEA = 0.022 (90% CI: 0.000, 0.126; TLI = 0.995 and CFI = 1.000, χ^2^ = 1.241) (*p* = 0.265, df = 1).

### Direct and indirect effects

Negative oceanic feelings were directly associated with personality organization (*β* = 0.37; *p* < 0.001). Additionally, personality organization was significantly linked to schizotypy (*β* = 0.51; *p* < 0.001), connectedness (*β* = 0.17; *p* < 0.001), and general religiosity (*β* = 0.10; *p* = 0.009). Negative oceanic feelings also had a direct effect on schizotypy (*β* = 0.15; *p* < 0.001) and reduced the sense of connectedness (*β* = −0.09; *p* = 0.014). Furthermore, negative oceanic feelings had a negative impact on general religiosity (*β* = −0.12; *p* = 0.004). In contrast, positive oceanic feelings showed a strong positive effect on connectedness (*β* = 0.68; *p* < 0.001) and general religiosity (*β* = 0.59; *p* < 0.001), and a small effect on schizotypy (*β* = 0.15; *p* < 0.001).

**Figure 2:**
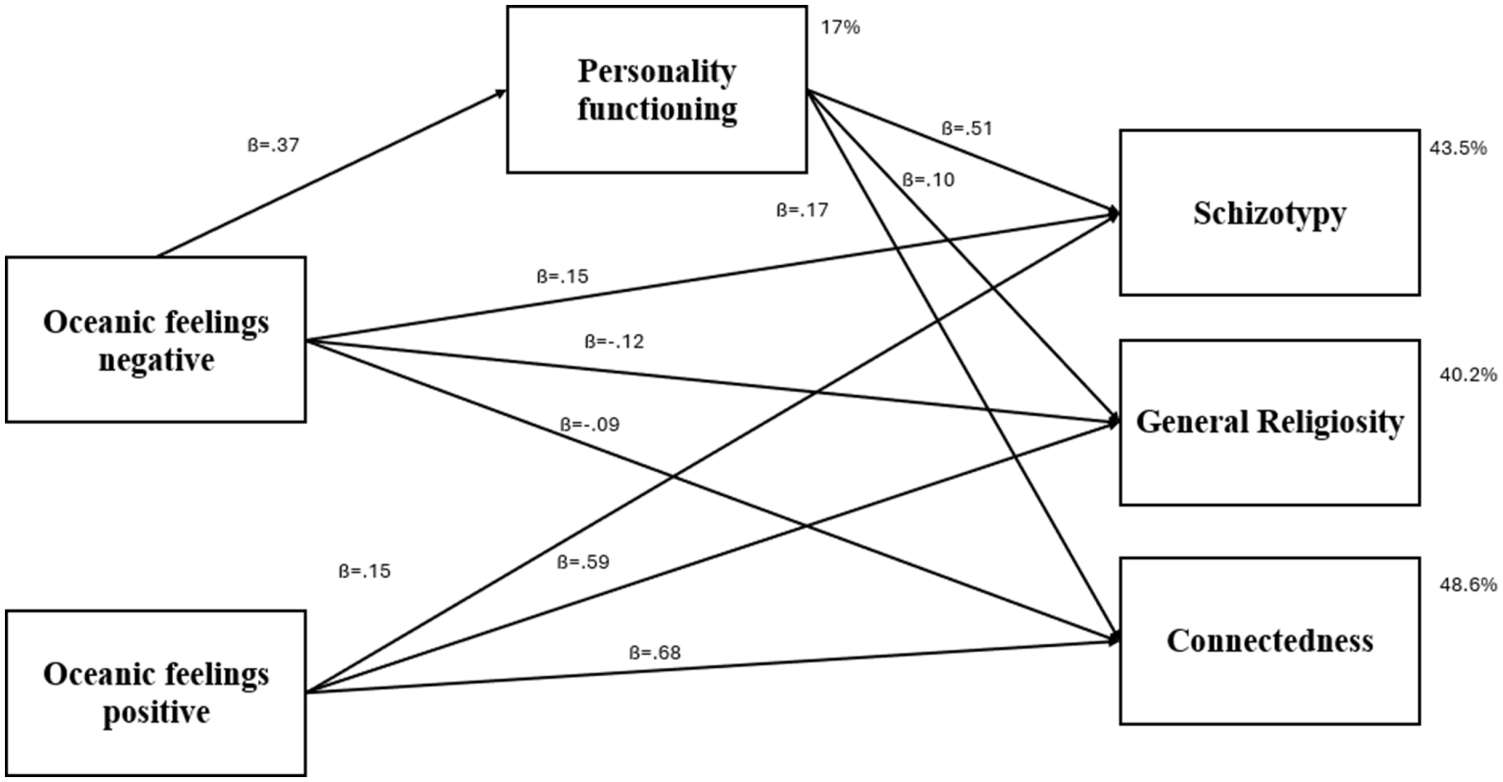
Mediated path analysis, controlled by age. Oceanic feelings and Schizotypy, General Religiosity and Connectedness were model as correlated, however, the correlations are omitted in this figure for better clarity.

Bootstrapping analysis revealed that the relationship between negative oceanic feelings and schizotypy was fully mediated by personality organization (*β* = 0.11, CI = 0.080, 0.144, *p* < 0.001). Additionally, the relationship between negative oceanic feelings and general religiosity was also fully mediated by personality organization (*β* = 0.05, CI = 0.012, 0.091, *p* < 0.011). In contrast, the association between negative oceanic feelings and connectedness was only partially mediated by personality organization (*β* = 0.07, CI = 0.036, 0.105, *p* < 0.001). The final model is illustrated in Figure 2. The path model was able to explain a total of 17.0% of the variance in personality organization, 40.2% of the variance in general religiosity, 48.6% of the variance in connectedness and 43.5% of the variance in schizotypy.

## Discussion

This study explored the relationship between oceanic feelings, schizotypy, personality organization, general religiosity, and connectedness, aiming to clarify oceanic feelings’ role in predicting schizotypy and spirituality during confluence experiences. Notably, positive oceanic feelings strongly enhanced connectedness and general religiosity, highlighting their affective foundation in spirituality and religiousness (Van Cappellen et al., 2014). In religious contexts, positive oceanic feelings may resemble experiences of God, akin to Christian mysticism as described by Meister Eckhart, involving a unifying “becoming one” experience where the self appears to dissolve (James, 1902). These mystical experiences closely parallel the oceanic feeling. However, as this study focused on a specific conceptualization of personality organization, different results might have emerged if alternative models or personality scales had been used.

Positive oceanic feelings were not significantly linked to personality organization, which could suggest that positive oceanic feelings are independent of personality organization. This might indicate that positive oceanic feelings occur in both mentally healthy individuals and those with psychotic disorders, reflecting the brain’s fundamental capacity for spiritual experiences (Fenwick, 2010). However, spiritual experiences likely emerge from a combination of factors, including cognitive, cultural, and contextual influences. In contrast, personality organization has a close relationship to negative oceanic feelings.

Negative oceanic feelings had a small but significant negative impact on connectedness and general religiosity. This finding could be interpreted as reflecting a mismatch of the experience with the predominantly positive Christian conception of God held by most participants, making these feelings harder to integrate. Rudolf Otto’s concepts of “mysterium fascinosum” (fascinating mystery) and “mysterium tremendum” (terrifying mystery) suggest that negative oceanic feelings might also be interpreted as divine encounters within the framework of “mysterium tremendum” (c.f. Schlamm, 1991). It was also shown that negative oceanic feelings were positively correlated with connectedness, but negatively related in the path model.

This study highlights the mediating role of personality organization. The path model showed that personality organization acts as a mediator for negative oceanic feelings. Personality organization fully mediated the relationship between negative oceanic feelings and schizotypy and between negative oceanic feelings and religiosity, and partly mediated the relationship between negative oceanic feelings and connectedness. In contrast, positive oceanic feelings were not mediated by the personality organization, which did not confirm hypothesis 2. The results of this study showed that positive oceanic feelings are experienced independently of personality disorganization and strongly correlated with religiosity and connectedness, while personality disorganization affects negative oceanic feelings and fully mediates their relationship with schizotypy and religiosity.

When trying to find answers from the clinical perspective, in the case of a low value of personality organization the boundaries between self and other in everyday experience are clear and the reference to the self reassured. Blurred boundaries between self and other may occur during experiences of intensified positive emotions, such as joy and lust—for instance, during dance, intimate moments, or sexual experiences—as well as during intensified negative emotions, such as grief or sadness. The results of this study indicate that positive oceanic feelings are part of a physiological spectrum of spiritual interactions with oneself and with the outer world with no significant influence by elements of personality organisation. Positive oceanic feelings constitute of emotionally positive or neutral experiences, while negative oceanic feelings reflect distinctly negatively valanced experiences.

With increasing levels of personality disorganization, the boundaries between self and other in everyday experiences become less distinct. Thoughts and feelings may feel less anchored to a clear sense of self, leading to greater ambiguity in distinguishing one’s own emotions and perceptions from those of others (see e.g., Nagel, 1974 for an enhanced philosopical discussion). The data from this study aligns with clinical observations suggesting that lower levels of personality organization are associated with a heightened vulnerability to schizotypal traits, such as ideas of reference, odd beliefs, magical thinking, paranoid ideation, and social isolation. This effect appears particularly pronounced when negative oceanic feelings are present, potentially contributing to an increased prevalence of schizotypy. This could explain how elements of personality organization influence the path of negative oceanic feelings and contribute to the manifestation of schizotypal symptomatology as well as to the development or absence of religiosity or certain elements thereof.

Strengthening personality organization may be key to managing negative oceanic feelings, offering insights for psychotherapy and spiritual counselling. Therapy focused on enhancing self-structure could potentially alleviate negative feelings and help integrate them constructively (Kernberg, 1984, 2019). Stabilizing ego boundaries, understood as reinforcing the distinction between self and others or the external world, has been suggested as particularly effective for individuals with intense spiritual or mystical experiences. Mindfulness-based interventions emphasizing self-awareness and body awareness could support this process without destabilizing personality (Tschacher and Munt, 2013). Nevertheless, further research is needed to explore the effectiveness of such interventions in different clinical and non-clinical populations.

### Limitations and future perspectives

These different mechanisms raise exciting questions for future research: Why is the effect of negative oceanic feelings explained by personality organization, while positive oceanic feelings are not? Could these be different psychological processes?

A possible limitation of this study is that positive and negative oceanic feelings may stem from distinct emotional constructs, potentially influencing their relationship with personality organization in different ways. Furthermore, an emphasis on assessing the frequency and duration of positive and negative oceanic feelings could shed a light on their connection with elements of personality organization. Another possible approach for further research would be to use moderation analyses. The sample, drawn from the general population, is predominantly highly educated and limited to German-speaking countries, restricting the generalizability of the findings. Data collection relied on self-reporting, which may introduce biases such as social desirability or memory errors. Although the final model demonstrated good fit indices after removing the non-significant pathway, the upper limit of the RMSEA confidence interval slightly exceeded the threshold for excellent fit (RMSEA = 0.022; 90% CI: 0.000, 0.126). This limitation should be considered when interpreting the overall fit of the model. Additionally, the study’s cross-sectional design precludes causal inferences.

While the Oceanic Feeling Scale (OFS) was applied using a randomized sample split, future studies should further assess its psychometric robustness in diverse populations to strengthen its generalizability. While PCA was used in the initial validation of the Oceanic Feeling Scale (OFS), we acknowledge its limitations in distinguishing shared and unique variance. Future studies will employ Exploratory Factor Analysis (EFA) and Exploratory Graph Analysis (EGA) to further refine the scale’s structure and ensure greater theoretical and statistical robustness.

Longitudinal studies are needed to examine the long-term effects of oceanic feelings on personality development and psychological or religious well-being. The link between positive and negative oceanic feelings and primary emotions presents intriguing directions for future research. Specifically, exploring connections to subcortical structures like the periaqueductal grey (PAG), central to emotional processing, could enhance the understanding of the neurobiological mechanisms underlying spiritual and psychotic states (Ferguson et al., 2021). And measure of personality disorganization may only reflect negative processes within personality, rather than healthy personality processes.

This study explored the link between oceanic feelings and schizotypy but did not include clinical schizophrenia patients or patients with other mental illnesses, such as dissociative disorders. Comparing individuals with psychedelic experiences or regular spiritual practitioners with the general population may reveal important differences. Such comparisons could help clarify whether certain traits, such as increased openness to experience or cognitive flexibility, contribute to the adaptive or maladaptive processing of oceanic feelings—potentially offering insights into mechanisms relevant to schizophrenia. Additionally, the cultural dimension of religiosity and upbringing in religious education should be considered when examining the connection between positive oceanic feelings and general religiosity. Future research could explore the impact of these feelings on religiosity across cultures with varying levels of religious education and upbringing. Additionally, future studies should explore how oceanic feelings, impact individuals’ faith, spirituality, and the image of God over the long term. In conclusion, some people experience ‘sweet delight’, others ‘endless night’. Our findings reveal that personality organization links negative oceanic feelings to spirituality and schizotypy. Negative oceanic feelings are mediated by personality organization. Positive oceanic feelings, on the other hand, act independently and correlate positively with connectedness and religiosity.

## Data Availability

The raw data supporting the conclusions of this article will be made available by the authors, without undue reservation.
